# Intestinal Anaemia

**Published:** 1913-11

**Authors:** 


INTESTINAL ANAEMIA. Prof. M. Loeper, Paris. Anaemia is not rare among patients with enteritis, either acute or chronic, whether the patients be young or adult. This anaemia is usually met with in certain cases of choleriform enteritis when microscopical examination shows the presence of bacilli resembling colib, paratyphoid, perfringens and enterocolic bacilli, and it may rapidly fall to 2,500,000 or even 2,000,000 red corpuscles. It is rather frequent in torpid intestinal conditions, muco-membranous enteritis, intestinal dyspepsia, typhlatony or typhlectasis, and then occurs in intermittent attacks appearing at the same time as an increase in the intestinal symptoms. This anaemia, connected both with hypohaemataemia and with hypo-haemoglobinaemia, urobilinhaemia, but not by choluria; the spleen is often hypertrophied and there may also be an increase in the of the liver. One may easily understand why anaemias which have their origin in the liver with intestinal troubles and abdominal pains should be often mistaken for liver attacks since both are characterised by the same discoloration of the skin and by an almost exactly similar localisation of the pains. Vomiting, however, as well as the pains spreading towards the shoulders and the urinary pigments are generally absent, and the condition is improved not by treating the liver but by treating the intestine, a point of the utmost importance. The examination of the stools reveals a slight insufficiency in the transformation of protein; the bacteria most frequently found are, as already stated, perfringens, enterococci and coliform bacilli. If we try to investigate the nature of these anaemias, we clearly realize that they are the result of a haemolytic process; still the resistance of the corpuscles is generally less, the auto-agglutination of the red corpuscles is absent but the increase in the haemolytic power of the serum is almost constant. The haemolysis is, therefore, the result of an exaggeration in the destructive power of the serum towards the red corpuscles, and not of a weakness of the red corpuscles themselves. If injected to rabbits, the serum of anaemic enteritic patients very often causes a diminution in the resistence of the corpuscles, and almost always a fall in the number of red corpuscles, this fall being much greater than with normal human serum.

The haemolytic substance is also hypotensive since these patients have always a tension below normal; it passes in the urine since the sediment of the urine experimentally produces hypotension and anaemia. This substance, which is certainly organic, seems to be produced in some cases by the ferments which are absorbed all along the alimentary canal; in other cases by bacteria, namely, perfringens and coli, the haemolytic action of which is well known; in other cases again by the hypersecretion of the intestinal cells or even by their destruction.

No doubt the bacteriolytic products play a prominent part in cases of acute enteritis; the products of a cellular origin, the cytolysins have an importance which seems to be greater in chronic cases. If extracts of the intestinal mucous membrane, and especially of the mucous membrane of the ileon and colon, are injected to animals, a distinct anaemia is produced; and the haemolysing action of these extracts is increased by addition of pancreatic ferments. Therefore the absorption by the inflamed human intestine of pancreatic ferments and of intestinal products is an important factor of haemolysis. In its normal condition the liver prevents the haemolysing action of all these substances, but its stopping function is greatly disturbed in enteritis.

Of the above expose leads to the following practical rules in the treatment of enterogenous anaemia: disinfection of the intestine, even with lactic bacteriotherapy and aperients in small repeated doses; strengthening of the powers of the liver by salts of magnesia; increase in the resistance of the blood by lipoids and regeneration of the blood by calcium and iron products.

